# The atmospheric electrical index for ENSO modoki: Is ENSO modoki one of the factors responsible for the warming trend slowdown?

**DOI:** 10.1038/srep24009

**Published:** 2016-04-04

**Authors:** Madhuri N. Kulkarni, Devendraa Siingh

**Affiliations:** 1 Indian Institute of Tropical Meteorology, Dr. Homi Bhabha Road, Pashan, Pune 411008, State Maharashtra, India

## Abstract

Like the southern oscillation index (SOI) based on the pressure difference between Tahiti (17.5°S, 150°W) and Darwin (12.5°S, 130°E), we propose the new atmospheric electrical index (AEI) taking the difference in the model calculated atmospheric electrical columnar resistance (*R*_c_) which involves planetary boundary layer height (PBLH) and aerosol concentration derived from the satellite measurements. This is the first non-oceanic index capable of differentiating between the conventional and modoki La Niña and El Niño both and may be useful in the future air-sea coupling studies and as a complementary to the oceanic indices. As the PBLH variation over Darwin is within 10% of its long term mean, a strong rise in the *R*_c_ over Darwin during the modoki period supports modoki’s connection with aerosol loading. Our correlation results show that the intensity of El Niño (La Niña) event is almost independent (not independent) of its duration and the possibility of ENSO modoki being one of the factors responsible for the warming trend slowdown (WTS).

There are various studies in atmospheric electricity showing an ENSO (El Niño and Southern Oscillations/La Niña) signal in global lightning and in the global atmospheric electric circuit (GAEC)^1^,^2^,^3^,^4^,^5^,^6^,^7^. The response of GAEC to global temperature has also been reported on various time scales^1^,^8^,^9^,^10^,^11^,^12^. The positive relationship between lightning (regional and global) and surface air temperature (SAT) (regional and global) on the ENSO time scale have been reported by many researchers^1^,^13^,^14^. From global measurements of SAT^4^,^15^ we see that all tropical regions warm during El Niño and cool during La Niña events.

In the present work, we consider two types of ENSO namely the conventional or the Eastern Pacific (EP) ENSO and the modoki or the Central Pacific (CP) ENSO. We develop a new method based on the newly constructed Atmospheric Electrical Index (AEI) and the Southern Oscillation Index (SOI)/Oceanic Niño Index (ONI) together for the characterization of the ENSO events. The SOI and the AEI is calculated for each month using the standardized difference in the pressure (p) and in the columnar resistance (*R*_c_) between Tahiti (17.5°S, 150°W) and Darwin (12.5°S, 130°E) respectively.

During the EP El Niño, the maximum of sea surface temperature anomaly (SSTA) lies in the EP away from the international dateline and that during the CP El Niño it lies in the vicinity of the dateline^16^,^17^. Similarly, when strong cold anomalies appear near the international dateline along with warm SSTA in the far EP, it is called as the La Niña modoki or CP La Niña^18^,^19^.

The problem of interference of the conventional and modoki signals and further elimination of the modoki signal while using different SST indices has been pointed out and an improvement has been suggested^20^ in the El Niño modoki index (EMI)^16^ used for differentiating between the EP and CP El Niño events. The SST indices are not useful in differentiating between the EP and CP La Niña as significant cold SSTA in the CP near the international dateline are found during both the types of cold events. Distinguished patterns of La Niña modoki have been obtained in the sea surface height (SSH)^19^ and sea surface salinity^21^ data analysis and a new SST index EMI2 based on the SSH analysis has been constructed. Both of these methods involve the oceanic parameters. Here, we suggest a non-oceanic index involving aerosol concentration for the first time and further discuss its variation with heat variability.

The main aerosol sources during ENSO are the presence of fires, biomass burning^22^,^23^,^24^ and an increase in the concentration of wind driven dust particles^25^,^26^ in a dry environment: an effect of subsidence of air over the region. Aerosol generation is also associated with changes in the Walker circulation as the changes in surface wind modulate the concentration of sea salt particles in the atmosphere. Climate events such as ENSO modulate aerosol emission sources, composition, transport pathways, types and concentration by modulating atmospheric dispersion characteristics^27^. As the *R*_c_ depends upon the aerosol concentration and aerosol distribution in a column due to vertical convection^28^, it is a proper atmospheric electrical parameter in the GAEC as a partial measure of ENSO-aerosol mechanism. Further, all the circulation systems are governed by the heating of the surface and the atmosphere causing changes in the locations of the high and low pressure regions which can cause changes in the *R*_c_. Thus, it is expected that similar to the pressure index SOI, the AEI may be useful for the characterization of the ENSO events.

## Method

The following expressions are used to calculate the AEI^29^,^30^


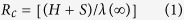


where *H* is the planetary boundary layer height (PBLH), *S* is the scale height of conductivity (=10 km/ln10)^31^ and *λ*(∞) is the average atmospheric electrical conductivity of the planetary boundary layer which depends upon the environmental aerosol and small ion concentrations. In (1), the required data parameters are PBLH, aerosol optical depth (AOD) and the profiles of cosmic ray ionization. The procedure to calculate the *R*_c_^30^ is given in the Supplementary Information.

Monthly PBLH data for Tahiti and Darwin for the period January 1980 to December 1992 and August 1996 to December 2011 used here are those produced by the Goddard Earth Observing System version 5 (GEOS-5) Modern Era Reanalysis for Research and Applications (MERRA) model and are available for free download from the Giovanni MERRA monthly (2D) data collection (2/3 × 1/2 horizontal grids). Similarly, the data of atmospheric circulation patterns (geo-potential height at 500 hPa, sea level pressure (SLP), vertical velocity (ω) at 500 hPa and upward long wave flux at top of atmosphere) (Supplementary Information) have also been downloaded from Giovanni MERRA monthly (2D) data collection (2/3 × 1/2 horizontal grids) for Supplementary Figure S2.

Our data show that the PBLH over Tahiti and Darwin are mostly below 1000 m. An overestimate of 30% in MERRA PBLH^32^ gives rise to only 5 to 6% error in the calculated *R*_c_ from (1). The *R*_c_ value quoted^33^ from the conductivity profile is ~1.67 × 10^17^ Ω m^2^ for PBLH ~2 km^34^ which gives *λ*(∞) ~4 × 10^−14^ mho m^−1^ from (1) above. This calculated *λ*(∞) is very well consistent with the profile value of conductivity^35^ at 2 km.

We use monthly AOD data for the period 1980–1992 and 1996–2000 generated by Total Ozone Mapping Spectrometer (TOMS) and those for the period 2001–2011 generated by Moderate Resolution Imaging Spectra Radiometer (MODIS) (Level 3, monthly collection 5.1 (051)) from the following web sites respectively:

ftp://toms.gsfc.nasa.gov/pub/nimbus7/data/aot/36.

http://gdata1.sci.gsfc.nasa.gov/daac-bin/G3/gui.cgi?instance_id = MODIS_MONTHLY_L3^37^.

for Tahiti and Darwin to derive aerosol concentration (*Z*)^38^.

As there is a data gap from January 1993 to July 1996 and few isolated missing data in the monthly PBLH time series, we follow the procedure for filling up the data gaps in the calculated *R*_c_ series^39^.

We use the monthly SLP data of Tahiti and Darwin^40^ for the base period 1980–2011 from the National Centers for Environmental Prediction (NCEP) to calculate the monthly SOI. Following the method adopted by the Bureau of Meteorology, Australian Government: (http://www.bom.gov.au/climate/glossary/soi.shtml)^41^, the SOI is calculated using the pressure difference between these two sites for each month. Similarly, the AEI is calculated using the difference in the columnar resistance between them for each month as given below:


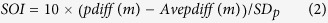



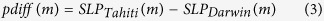



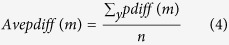


where m, y, n are the month, year and the number of years respectively and multiplication by 10 in (2) is a convention.

Similarly,


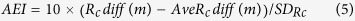



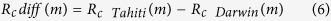



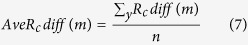


In the above equations (2, 3, 4, 5, 6, 7), *pdiff*(*m*) (*R*_c_ *diff*(*m*)) is the difference in pressure (columnar resistance) between Tahiti and Darwin for a particular month. *Ave pdiff*(*m*) (*Ave Rc diff*(*m*)) is the long term mean of *pdiff*(*m*) (*R*_*c*_*diff*(*m*)) and *SD*_*p*_ (*SD*_*Rc*_) is the long term standard deviation of *pdiff*(*m*) (*R*_*c*_*diff*(*m*)) respectively.

The SOI and the AEI are calculated for individual months from (2–4) and (5–7) respectively to identify the EP and CP ENSO events. A statistically significant positive correlation ~0.82 (p < 0.0001) and ~0.63 (p < 0.0001) has been obtained between the filtered monthly *R*_c_ and SLP series for Tahiti and for Darwin respectively on the ENSO time scale of 2–7 years (Figure S1). The ONI data have been used from the web site of the Climate Prediction Center (CPC) given below to verify the strength of the event deduced from the AEI:

http://www.cpc.noaa.gov/products/analysis_monitoring/ensostuff/ensoyears.shtml^42^.

Monthly SAT data from Goddard Institute for Space Studies (GISS), NASA have been used to carry out the correlation analysis between the constructed AEI and the SAT data over Darwin to study the AEI’s association with the heat variability.

## Results and Discussion

Table 1 displays most of the warm and cold events during the period 1980–2011^17^,^43^,^44^,^45^ considered in the present analysis along with their duration, type, the calculated *R*_c_ over Tahiti and Darwin, and the calculated AEI and SOI. Each AEI in Table 1 is the average of all monthly AEIs during the respective period of the event. Similarly, other parameters in Table 1 are also averaged during the respective period. The SOIs are negative for El Niño and positive for La Niña. We can see in Table 1 that the CP events are characterized by the negative AEI and the EP events by the positive AEI. There are various criteria in literature to identify the CP and the EP events^16^,^19^,^21^. In literature, the identification of the La Niña event of 1998 is ambiguous of being CP^16^ or EP^19^.

### Special cases of ENSO and ENSO modoki events

The ambiguity (whether EP or CP) of the La Niña event of 1998 is clearly reflected in the calculated AEI magnitude for the event which is very low positive as compared to other La Niñas. The La Niña of 1998 was different on the basis of convection and circulation characteristics^46^. This is reflected in the low positive AEI and less decrease in the *R*_c_ over Darwin as compared to other EP cold events. The La Niña of 1998 was preceded by the EP El Niño of 1997. From Table 1 we see that there is a decrease of 26% in the *R*_c_ during this EP El Niño event as many parts of Australia received rainfall including Darwin. For the La Niña events of July 1998-December 1999 and January 2000-February 2001, according to the analysis of Bureau of Meteorology, Australian Government:

(http://www.bom.gov.au/climate/enso/lnlist/)^47^, there were rainfalls over Australia up to March 1999 including Darwin and considerably drier conditions from April 1999 to September 1999 but wet conditions over Darwin. This can cause decrease in the *R*_c_ over Darwin during the period July 1998-December 1999. La Niña conditions appeared again from October 1999-May 2000. June 2000 to September 2000 was again a dry season for Australia including Darwin in which aerosols can increase. La Niña conditions appeared again from October 2000 to March 2001 giving above average rainfall over Darwin. Due to this above average rainfall over Darwin, the resultant increase in *R*_c_ is only 6% (event 9, Table 1) which is less than other CP La Niña events in Table 1.

The EP El Niño of 1982–1983 was one of the intense events and the SSTAs were spread from the CP up to the EP. This is reflected in a small positive value of the AEI and justifies the finding that El Niño modoki pattern of SSTA is a part of the El Niño evolution^16^,^48^. As the event is redundant in the CP and then the SSTAs started spreading over the EP, the initial temporary increase in the aerosol concentration over Darwin due to dry conditions disappeared. Further, changes in the location of convection and circulation pattern caused the *R*_c_ over Darwin to decrease and made it to an extent greater than that during the EP event. This caused AEI to be of small positive magnitude.

A very large increase in the *R*_c_ for event 6 in Table 1 may be attributed to the volcanic eruption in the Indonesian region during 1990–1991 and a severe volcanic eruption (Mount Pinatubo) occurred in Phillippines in 1991 which created global effects. Similarly, the volcanic eruptions in Papua New Guinea during 2004–2007 could have caused the excessive increase in the *R*_c_ for events 11, 12 and 13. Year 2004–2005 was a drought year for Australia with the strong anomalies of maximum temperature, pressure and rainfall causing excessive dryness. During June 2005, areas inland from Australia’s Great Dividing Range in Victoria, New South Wales and southeast Queensland, as well as eastern South Australia, received good rainfall^49^ but northern Australia was completely dry. So, there was no wet deposition of aerosols accumulated during the CP El Niño of 2004–2005. On the other hand, there was a large deficit of rainfall and continuing dry conditions. This is reflected in a very large increase in the *R*_c_ (~63%) (event 12, Table 1).

Except the EP La Niña of 2010–2011 (event 16, Table 1), the other EP La Niñas in Table 1 follow the EP El Niños. The EP La Niña of 2010–2011 follows the CP El Niño (event 15, Table 1) of 2009–2010. This caused small decrease in *R*_c_ over Darwin compared to other La Niñas as during CP El Niño, there is a large increase in aerosol concentration over Darwin. Also, there was a volcanic eruption of Rabaul volcano during 2010 in Papua New Guinea. The La Niña event of 2010 was from 2010–2012 having two peaks over the successive summers of 2010–2011 and 2011–2012. 2010 was the second wettest calendar year on record for Australia. During this event, there was widespread flooding and unusual rain events in the dry season over Australia which caused only 6% reduction in *R*_c_ which is less compared to other EP La Niña events in Table 1.

### The *R*_c_ changes Over Tahiti and Darwin during the EP and CP ENSO

The changes in convection on account of SSTA modulate atmospheric transport^50^ and affect planetary wave activities in the tropics which further influence tropical upwelling^51^. Thus, different SSTA pattern in two types of El Niño events would lead to different transport processes between troposphere and stratosphere. A recent study shows that El Niño modoki modulates the tropical upwelling in which tropospheric air and constituents enter the stratosphere. This upward motion in the tropical stratosphere leads to an adiabatic cooling. This affects the tropical columnar ozone ultimately producing changes in the circulation^52^,^53^,^54^.

Considering all the CP events in Table 1, the average increase in the *R*_c_ over Tahiti is about 1% during the CP El Niño as well as during the CP La Niña. Over Darwin we have a 43% increase in the *R*_c_ during the CP El Niño and 32% increase during the CP La Niña period.

During El Niño, the aerosol loading increases due to forest fires in the Indonesian region^55^ and dry environment over the Australian region (Figure S2a,b,e,f,j) causing an increase of the dust particles in the atmosphere. Both of these factors contribute towards increase in the aerosol loading over Darwin. During the CP or modoki events, due to change in the SSTA pattern, there is a shift in the convection center causing modulation of the atmospheric circulation pattern and in effect modulation of aerosol pathway trajectories (e.g. Figure S2a,b).

The increase in aerosol loading over Darwin during the El Niño modoki period may be attributed to the increase in aerosol concentration on account of fires in the savanna of North Australia in the vicinity of Darwin, prevailing high pressure over the Australian region causing subsidence (Figure S2a), the changes in the tropical upwelling and the role of the subtropical ridge in causing excessive dryness by inhibiting the frontal activity^49^ which brings moisture to the south of the continent. Also, the aerosols injected at higher levels due to volcanic eruptions in the Indonesian regions can subside over Australia due to prevailing high pressure. Sometimes, during the EP El Niño there is precipitation in autumn over Australia which may cause a wet deposition of aerosols (Figure S2m) but during the CP El Niño, complete dry and drought conditions (Figure S2n) prevail^18^ and disposal of aerosols by wet deposition may not occur. This may cause large AOD during the CP El Niño period over Australia. It has been shown^19^ from the composite SST analysis for 29 years from 1983–2011 that during the El Niño period, negative SSTAs prevail near Darwin. The situation is similar over Tahiti. For the CP El Niño event of 2002, the descending branch of atmospheric circulation was over the Australian maritime continent^56^. On the other hand, in the vicinity of Tahiti, there is an ascending branch just west of Tahiti and a descending one to the east. This creates a clockwise circulation over Tahiti and may cause only a small change (~ +1%) in the *R*_c_ (Figure S2b).

During the EP El Niño, the conditions over Australia are not excessively dry due to change in the location of subsidence and sometimes occurrence of precipitation causing decrease in aerosol concentration over Darwin. For the EP El Niño event of 1997, the descending branch of atmospheric circulation was not over the Australian continent but it was centered just east of Australia at 150° E^56^ which is also seen in our composite analysis (Figure S2i). This shift in the location of the descending branch and the intermittent precipitation over Australia can cause the decrease in AOD over Darwin during the EP El Niño.

During the La Niña period, the composite SST analysis^19^ shows warm SSTA (0 to 0.2) near Darwin and as Tahiti is flanked by warm SSTA, there exists opposite SSTA on both the sides of Tahiti (0 to −0.2 and 0 to 0.2). Thus, there is convection near Darwin and anticlockwise circulation over Tahiti (Figure S2c,g,h,k and l).

Generally, a La Niña event occurs at the end of an El Niño event.

During La Niña modoki, northwest Australia (Figure S2d,p) receives rainfall^18^. There is a large increase in aerosol concentration during El Niño period which can partially settle down due to wet deposition during La Niña modoki. This might have caused an average ~32% increase in the *R*_c_ over Darwin during the CP La Niña: 11% less than that during the El Niño modoki. Though, there is precipitation during the EP and CP La Niña, the impact of CP La Niña is from the northwest i.e. in the vicinity of Darwin (Figure S2p) and the savanna region in north Australia. So, the air over Darwin during the CP La Niña may have more aerosol content than that during the EP La Niña. The increase in *R*_c_ over Darwin during the CP La Niña may partly be attributed to the entering air mass content and partly to the increase in sea salt particles in the atmosphere due to strengthened Walker Circulation (ascending branch at about 110°E) causing bursting of sea bubbles due to wind stress over the maritime continent. Tahiti is flanked by warm SST during the CP La Niña and by cold SST during the CP El Niño. This may cause no distinct change in the *R*_c_ over Tahiti.

The impact of EP La Niña is from the southeast (Figure S2o). A wet deposition of aerosols and change in the impact region contribute to the reduced AODs during the EP La Niña. Darwin is towards northwestern side of Australia while the impact of EP La Niña concentrates in southeast and south Australia.

If the CP El Niño is followed by a La Niña event, it can cause large deposition of aerosols to ground (e.g. event 16), thereby reducing their concentration in the air.

In Table 1, the PBLH are within ± 10% of its long term mean. This implies when the PBLH over Darwin is not appreciably different from its long term mean, a strong increase in the AOD (up to 55% in case of the El Niño modoki and up to 46% in case of the La Niña modoki) or *R*_c_ over Darwin during the modoki period confirms modoki’s connection with aerosol loading.

### Identification of the EP ENSO and ENSO modoki using the AEI

**Figure 1:**
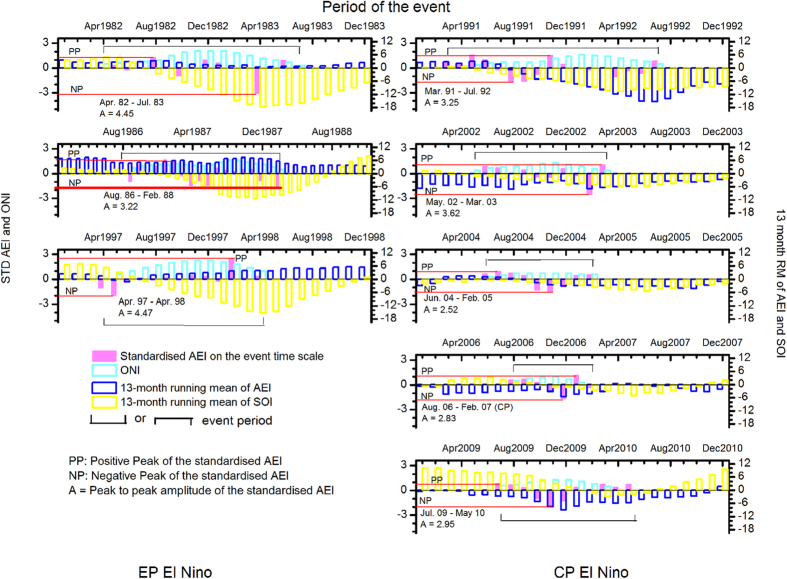
Variation of 13-month running mean of the SOI and the AEI from January of the starting year to December of the ending year for El Niño, the standardized AEI and the ONI during the event. This figure has been prepared using Origin 8.5 software.

**Figure 2:**
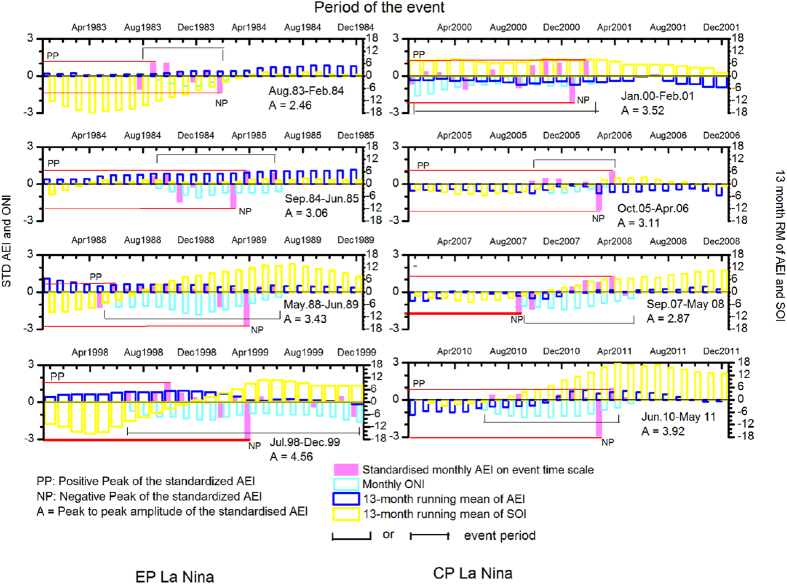
Variation of 13-month running mean of the SOI and the AEI from January of the starting year to December of the ending year for La Niña, the standardized AEI and the ONI during the event (the La Nina event of Jun 10 - May 11 in the right column bottom panel should be read in the left column (EP La Nina) of Figure 2). This figure has been prepared using Origin 8.5 software.

Figures 1 and 2 show variation of the AEI and SOI (13-month running mean (leading)) during the El Niño and La Niña periods respectively along with the standardized monthly AEI on the time scale of individual event and the corresponding monthly ONI during the event. The running mean is plotted for the period: from January of the starting year of the event to December of the ending year of the event. From Figs 1 and 2 we can notice the peculiar behavior of the AEI pattern during the El Niño and La Niña events i.e. they are in phase with the SOI for the CP El Niño and EP La Nina and in out of phase for the EP El Niño and CP La Niña. Generally, the EP and CP El Niño events are preceded by a trend of decreasing positive SOI and/or with increasing their negative magnitude.

Similarly, for the La Niñas, one can observe decreasing negative amplitude of the SOI and/or becoming more positive during the events (Fig. 2). Like the CP El Niño, the AEIs for the CP La Niña also appear negative indicating considerable increase in the *R*_c_ over Darwin. The peak to peak amplitude (*A*) of the standardized monthly AEI on the time scale of the event indicates the intensity of the event as shown in Figs 1 and 2 and in Table 2. A statistically significant correlation ~0.91 (−0.64) between *A* and the average ONI for the El Niño (La Niña) events is non-significant (significant) ~−0.11 (+0.75) when *A* is weighted by the duration (Figure S3). Thus, duration of the event plays role in the case of intensity of La Niña but not in the case of El Niño. The conditions are stable over Australia during El Niño due to existing high pressure. The atmospheric column over Darwin gets saturated with the trapped aerosols and there exists balance between the influx of aerosols from the upper levels and the outgoing of aerosols near the surface. Thus, the conditions within the column become independent of time. During La Niña the conditions are unstable over Darwin due to existing low pressure and such a balance may not exist. The changes in the *R*_c_ during ENSO over Tahiti are small compared to those over Darwin due to clean air over the eastern Pacific.

From Table 1 and Figs 1 and 2, negative SOI, negative AEI characterize the CP El Niño event and positive SOI, negative AEI characterize the CP La Niña event. Similarly, negative SOI and positive AEI characterize the EP El Niño and positive SOI and positive AEI characterize the EP La Niña.

We can see from (Table 1) and Figs 1 and 2 that the type of all 16 events is successfully identified as the EP or CP. Also, it is seen that generally, it may be possible to predict the type of the event few months before as the AEI pattern shows its polarity in advance of the starting month of the event. The exception for predicting the type occurs only when there is a global effect of volcanic eruption (event 6, 11 and 16, Table 1). Thus, out of these 16 events, the type of 13 events can be detected correctly few months in advance. Our calculations show more number of positive (negative) monthly AEI during the EP (CP) ENSO event irrespective of global effects of volcano. Identifying and predicting the type of event is very important as the CP El Niño causes opposite effects to the EP El Niño due to different warming region and a tri-polar SST pattern. ENSO modoki is a predictor for the tropical columnar ozone^49^ which plays a major role in the atmospheric warming and cooling effect.

It has been reported^57^ that internally generated variability dominates the variability of SAT on the multidecadal time scale in extratropics. This is again confirmed for the whole Northern Hemisphere^58^ too. The interannual variation of SST anomalies has been linked to persistent regional and global atmospheric anomalies^59^,^60^. Looking at our results in Figs 1 and 2 we see that aerosols play major role in deciding the type of the event. We know that reflecting and scattering aerosols create cooling effect while those absorbing create warming in the atmosphere. This atmospheric effect causing heat variability may cotribute to the changes in the ONI and global circulation as a coupling between the oceanic and the non-oceanic parameters. It has been reported^58^ that the dynamically induced SAT variability creates cooling effect opposite to that caused due to radiatively forced SAT changes on account of anthropogenic forcing i.e. warming due to green house gases. The main cause of dynamically induced SAT variability are North Atlantic Oscillation (NAO), Pacific Decadal Oscillation (PDO) and Atlantic Multidecadal Oscillation (AMO). Atmospheric circulations change due to anthropogenic forcing which further creates SAT variability. Thus, aerosol pathways are important in case of SAT variability. As our results show major changes in the *R*_c_ over Darwin compared to those over Tahiti, we use GISS SAT data (250 km smooth, 2° × 2° grid) over Dawin to study the relationship between the AEI and SAT over Darwin in the following two ways: (i) to study variability in both the series on multi-decadal time scale i.e. between the adjusted SAT^58^ and the AEI and (ii) to study the inter-annual variability in the AEI and SAT with the corresponding average conditions of the AEI and SAT independently during the total El Niño and La Niña period, only EP El Niño and EP La Niña period and only CP El Niño and CP La Niña period.

Similar analysis has also been done for the ONI series.

From the definition of the AEI when it becomes negative, the Rc over Darwin is higher than that over Tahiti indicating higher concentration of aerosols over Darwin as the PBLH variations are not appreciable. Further, the Rc variations over Tahiti are too low as compared to that over Darwin. So, it is not unreasonable to view the AEI variations in terms of variations in the aerosol concentration over Darwin. More and more negative AEIs indicate more and more build up of aerosols over Darwin. On the contrary, more and more positive AEIs indicate decrease of aerosols over Darwin.

Figure S4 shows the variations in the adjusted SAT over Darwin and the corresponding AEI anomaly. We get a high positive statistically significant correlation between the two series (Pearson’s correlation coefficient ~0.74 with p > |F| = 0). The series have nearly parallel trends but the variations are out of phase. An increase in the AEI corresponds to decrease in the SAT anomaly i.e. decrease in aerosol concentration over Darwin creates cooling effect in the adjusted SAT anomalies. Thus, the aerosols are creating warming effect in the adjusted SAT consistent with the global warming concept. Further, from Figure S4 the fluctuations in SAT from 2001 onwards are such that making the trend slower than that in the previous decade and therefore may be identified as the SAT hiatus period over Darwin. The corresponding hiatus we observe in the AEI anomaly series too. Similar features are seen in the AEI anomaly and ONI series with statistcally significant little lower correlation coefficient (~0.55, (p > |F|) < 0.05) (Figure S5).

Figure S6 shows the results of correlation statistics between the anomaly series of AEI and SAT (Darwin) after taking 13-months running mean of both the series. These inter-annual variations in the AEI show statistically significant positive correlation with the SAT anomaly over Darwin only in the case of EP ENSO (both warm and cold events). Our results do not show significant relationship in the case of CP ENSO. Similarly, Figure S7 shows the results for the anomaly series of AEI and ONI after taking 13 months running mean. In this case we get the significant correlation between the two series for the CP ENSO only. Thus, AEI anomaly shows significant correlation with the SAT anomaly over Darwin during the EP events and with the ONI during the CP events. The possible explanation may be given in terms of the difference in type of aerosols during the EP and CP events. The SAT anomalies during the EP ENSO may be governed mostly by a single type of aerosols i.e. either absorbing or reflecting-scattering. In this condition, the SAT anomaly may be proportional to the number concentration of aerosols over Darwin. On the other hand, if the aerosol population is not dominated by a single type and it contains absorbing as well as reflecting-scattering aerosols, both comparable, the resultant SAT anomaly will not be proportional to the total number concentration of aerosols due to their contradictory radiative effects. Table S1 shows the average anomalies of AEI, ONI and SAT during different periods. The average anomalies of AEI and SAT are positive during the EP ENSO. Thus, decrease of aerosols creates warming over Darwin. This may be attributed to the decrease in reflecting-scattering aerosols over Darwin during the EP ENSO.

As the increase in aerosols and the corresponding SAT anomaly over Darwin are not associated significantly in case of CP events probably due to mixed aerosol population, it may be one of the factors responsible for the occurrence of SAT hiatus. As the period 1980–2000 is not dominated by the CP ENSO, we do not observe warming trend slowdown (WTS) during this period. On the other hand, after 2000, most of the ENSO events are of CP type which may suppress intense SAT variations. During the period 1951–1970^61^ too, most of the events are identified as the CP events which may be responsible for the earlier SAT hiatus^58^ during 1940–1970. The first hypothesis of WTS is linked to natural variability i.e. the surplus heat absorbed by the climate system is stored in ocean and is not used for warming the land surface. Our present results show consistency with the above hypothesis in the case of CP events. The results obtained for the AEI-SAT variability over Darwin and the AEI-ONI variability are consistent with the above hypothesis with further specializing the type of ENSO as CP ENSO which may be one of the factors responsible for the occurrence of WTS.

As mentioned before, there is a close association between climate/environmental change/SAT and atmospheric electrical parameters. Since the ionospheric potential V_i_ acts as a global thermometer^1^, we have examined its role during one of the El Niño events during April 1982-Jul 1983 depending upon the availability of V_i_ data^11^ (Figure S8 and Table S2) We can see that the V_i_, resistive power dissipation due to Joule heating (V_i_ × I_t_) and the total air-earth current (I_t_) are in phase with the ONI showing a close association between the El Niño: a global climate event and the corresponding global atmospheric electric circuit. The link has been proposed^1^,^62^ as: Since the V_i_ is maintained by global thunderstorm activity and the electrified shower clouds, it serves as a measure of global temperature change. As the V_i_ and convection is driven by temperature differences/heat variability, the variation in V_i_ should be a measure of the temperature changes where the most deep convection occurs. As the ONI are for the tropical region near the equator and are the measure of global climate events, the V_i_ shows parallel variation with the ONI during the event.

Finally, the summary of the research outputs is given below:

The proposed AEI is the first non-oceanic index to characterize and possibly predict the type (conventional or modoki) of the El Niño and La Niña events. It is especially useful in the case of La Niña as the identification becomes difficult using SST indices. The peak to peak amplitude of the standardized AEI determines the intensity of the event. Our results show that duration of the event is a contributing factor for determining the intensity of La Niña but not for El Niño.

We find substantial increase in the columnar resistance over Darwin during the ENSO modoki and decrease during the EP ENSO. This may be attributed to the meteorological conditions over Australia and changes in the atmospheric circulation causing regional effects.

When the PBLH over Darwin is not appreciably different from its long term mean, a strong increase in the AOD/*R*_c_ over Darwin during the modoki period confirms the modoki’s connection with aerosol loading.

The main limitation of the AEI is not being as direct as other ENSO SST indices. On the contrary, this is the first index involving aerosol formally for distinguishing the EP and the CP ENSO. The scope of the present study is not limited only to the characterization of the ENSO event but also for the future multidisciplinary studies in aerosol, atmospheric electricity (e.g. global electric circuit) and meteorology (e.g. air-sea coupling).

We see from our simple analysis the possibility of CP ENSO being one of the factors responsible for the WTS. Further climate model studies may confirm this in the future.

## Additional Information

**How to cite this article**: Kulkarni, M. N. and Siingh, D. The atmospheric electrical index for ENSO modoki: Is ENSO modoki one of the factors responsible for the warming trend slowdown?. *Sci. Rep.* **6**, 24009; doi: 10.1038/srep24009 (2016).

## Supplementary Material

Supplementary Information

## Figures and Tables

**Table 1:** Calculated *R*_c_ and AEI with the corresponding satellite data of AOD and PBLH over Tahiti and Darwin.

No.	Type ofthe event	Period	PBLH (m)(Tahiti)	AOD_500_(Tahiti)	*R*_c_ × 10^17^ (Ω m^2^)(Tahiti)	% change inR_c_(Tahiti)	PBLH (m)(Darwin)	AOD_500_(Darwin)	*R*_c_ × 10^17^ (Ω m^2^)(Darwin)	% change inR_c_(Darwin)	AEI	SOI
1	EP El	Apr. 82-Jul.83	890	0.0839	1.44	−13	609	0.1274	2.20	−18	0.80	−14.21
2	EP La	Aug. 83-Feb.84	880	0.0773	1.39	−16	624	0.1105	1.90	−29	4.76	2.75
3	EP La	Sep.84-Jun.85	789	0.0987	1.60	−4	653	0.1075	1.91	−29	6.52	2.12
4	EP El	Aug.86-Feb.88	840	0.1030	1.62	−2	580	0.1055	1.82	−32	6.45	−8.38
5	EP La	May88-Jun.89	899	0.0942	1.50	−10	618	0.1028	1.90	−29	2.24	12.81
6	CP El	Mar. 91-Jul. 92	911	0.1318	1.97	+19	611	0.2078	4.11	+53	−11.15	−9.24
7	EP El	Apr.97-Apr. 98	868	0.0901	1.60	−4	663	0.1150	2.00	−26	3.90	−15.22
8	EP La	Jul.98-Dec.99	954	0.0986	1.57	−5	600	0.1394	2.40	−11	1.00	9.31
9	CP La	Jan.00-Feb.01	888	0.0952	1.59	−5	617	0.1736	2.85	+6	−3.08	8.12
10	CP El	May02-Mar.03	923	0.0906	1.47	−11	621	0.1922	3.54	+32	−7.98	−6.35
11	CP El	Jun.04-Feb.05	906	0.0919	1.50	−10	552.	0.1998	3.95	+47	−6.38	−6.78
12	CP La	Oct.05-Apr.06	825	0.1055	1.64	−1	670	0.2145	4.38	+63	−9.33	6.77
13	CP El	Aug.06-Feb.07	851	0.1314	1.94	+17	573	0.2283	4.04	+50	−5.21	−5.95
14	CP La	Sep.07-May08	810	0.1182	1.81	+9	610	0.1834	3.43	+28	−1.90	8.77
15	CP El	Jul.09-May 10	854	0.0916	1.49	−10	614	0.1851	3.58	+33	−4.98	−2.03
16	EP La	Jun.10-May11	889	0.1226	1.83	+10	588	0.1393	2.53	−6	4.33	17.48

The values in the columns denote average for the respective period of the event and % change in R_c_ is with respect to the average R_c_ for the base period.

Base period: 1980–2011, Average PBLH over Tahiti for the base period = 878 m, and over Darwin = 606 m.

Average R_c_ over Tahiti for the base period = 1.66 × 10^17^ Ω m^2^, and over Darwin = 2.68 × 10^17^ Ω m^2^.

Average AOD_500 nm_ over Tahiti for the base period = 0.103, and over Darwin = 0.147.

**Table 2:** Intensity of events (A) derived from the standardized AEI.

Event duration	Event type	Intensity of the event (A)
Apr. 82-Jul. 83	EP El	4.45
Aug. 86-Feb. 88	EP El	3.22
Mar. 91-Jul. 92	CP El	3.25
Apr. 97-Apr. 98	EP El	4.47
May 02-Mar. 03	CP El	3.62
Jun. 04-Feb. 05	CP El	2.52
Aug. 06-Feb. 07	CP El	2.83
Jul. 09-May 10	CP El	2.95
Aug. 83-Feb. 84	EP La	2.46
Sep. 84-Jun. 85	EP La	3.06
May 88-Jun. 89	EP La	3.43
Jul. 98-Dec. 99	EP La	4.56
Jan. 00-Feb. 01	CP La	3.52
Oct. 05-Apr. 06	CP La	3.11
Sep. 07-May 08	CP La	2.87
Jun. 10-May 11	EP La	3.92
