# Supplementary material for: The atmospheric electrical index for ENSO modoki: Is ENSO modoki one of the factors responsible for the warming trend slowdown?
Source: Sci Rep. 2016 Apr 4;6:24009. doi: 10.1038/srep24009 (PMC4819170; doi:10.1038/srep24009)
Supplement: Supplementary Information [file srep24009-s1.pdf]

Supplementary online Material for

The atmospheric electrical index for ENSO modoki: Is ENSO modoki one of the factors responsible for the warming trend slowdown?

Madhuri N. Kulkarni and Devendraa Siingh

Composite Atmospheric Circulation Patterns

Figure S2 (supplementary figures given below**)** includes composite atmospheric circulation patterns supporting our discussion in the manuscript. These are anomalies of the geo-potential height (GPH) at 500 hPa**,** vertical velocity (ω) at 500 hPa , sea level pressure (SLP) and upward long-wave flux at top of atmosphere. The climatology period for calculating the anomalies is from year 1980 to 2011. The anomaly of parameter 'P' at latitude 'i' and longitude 'j' is given by,

$$(P_{i,j}) = \text{average}\,(P_{i,j})\big|_{T1} - \text{average}\,(P_{i,j})\big|_{T}$$

where T1 is the total period of the events of a particular type and T is the total period 1980-2011.

For all the above parameters the means of the CP (modoki) and the EP (conventional) events are significantly different at statistical significance level $\alpha = 0.05$ using Student's t test.

Supplementary Table

Table S1: The average of ONI, AEI and SAT anomalies

Period	AEI anomaly	ONI	SAT anomaly over Darwin
Total El Niño	-0.236	0.988	0.375
Total La Niña	0.083	-0.926	0.236
EP El Niño	0.393	1.246	0.445
EP La Niña	0.333	-0.985	0.315
CP El Niño	-0.786	0.764	0.314
CP La Niña	-0.424	-0.807	0.074

Table S2: Measured ionospheric potential (V_i)[1] and the corresponding calculated atmospheric electrical parameters and the ONI during the El Niño event 1 of Table 1 (April 1982-July 1983).

Data Point No.	Month and Year during the event	V_i[1] (measured) KV	Calculated R_c at Tahiti (Ω m^2 × 10^{-17})	Calculated total air-earth current density (I_t) (pA m^{-2})	Power generated due to joule heating (Joules sec^{-1}×10^7)	ONI (°C)
1	May 1982	249	1.547	1.609	4.006	0.6
2	Aug.1982	282	1.667	1.692	4.771	1
3	Feb.1983	316	1.307	2.418	7.641	1.8
4	Mar.1983	251	1.391	1.804	4.528	1.5
5	Apr.1983	207.5	1.275	1.627	3.376	1.2

Supplementary Figures

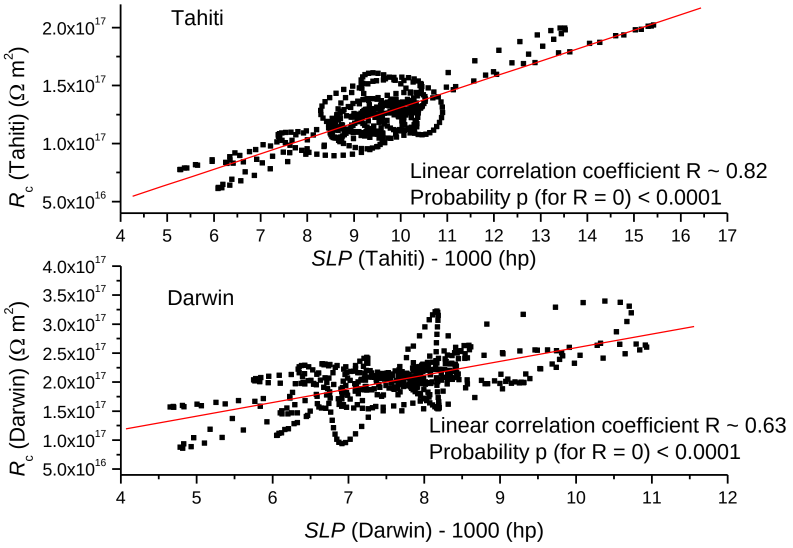

Figure S1: The scattered plots showing the correlation statistics between the filtered series of the columnar resistance (R_c) and the Sea Level Pressure (SLP) at Tahiti and Darwin on the ENSO time scale of 2-7 years using the FFT band pass filter. This plot has been made using Origin 6.1 software.

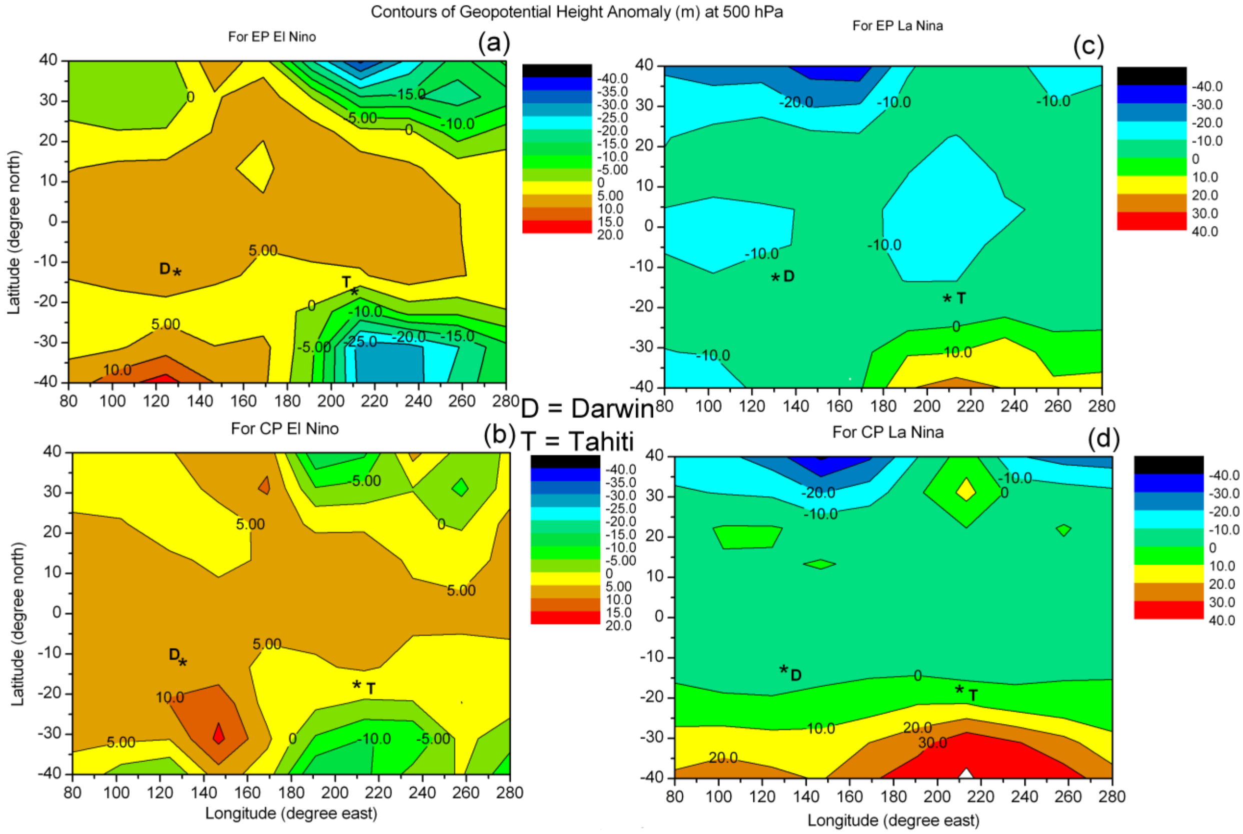

Figure S2: Composite atmospheric circulation patterns. Origin 8.5 software has been used for making these plots.

Figure S2 continued…………..

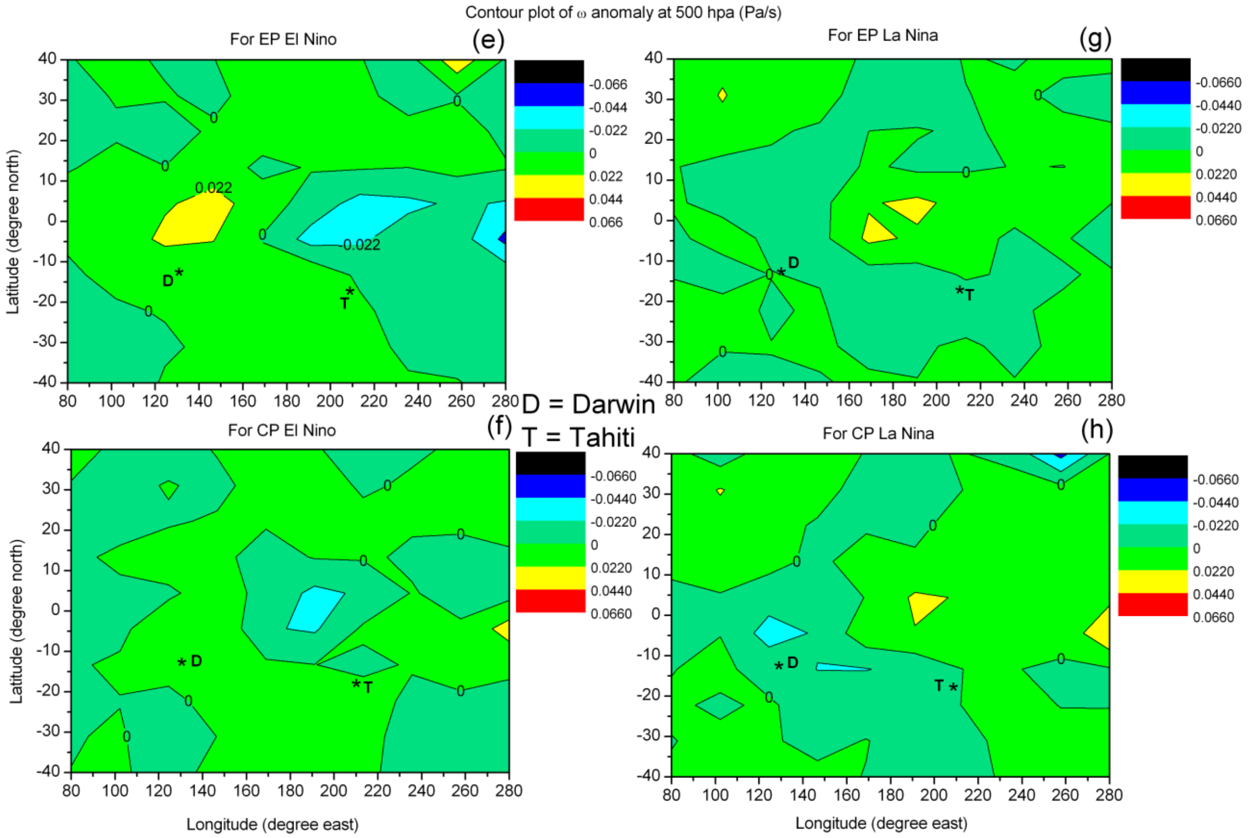

Figure S2 continued…………..

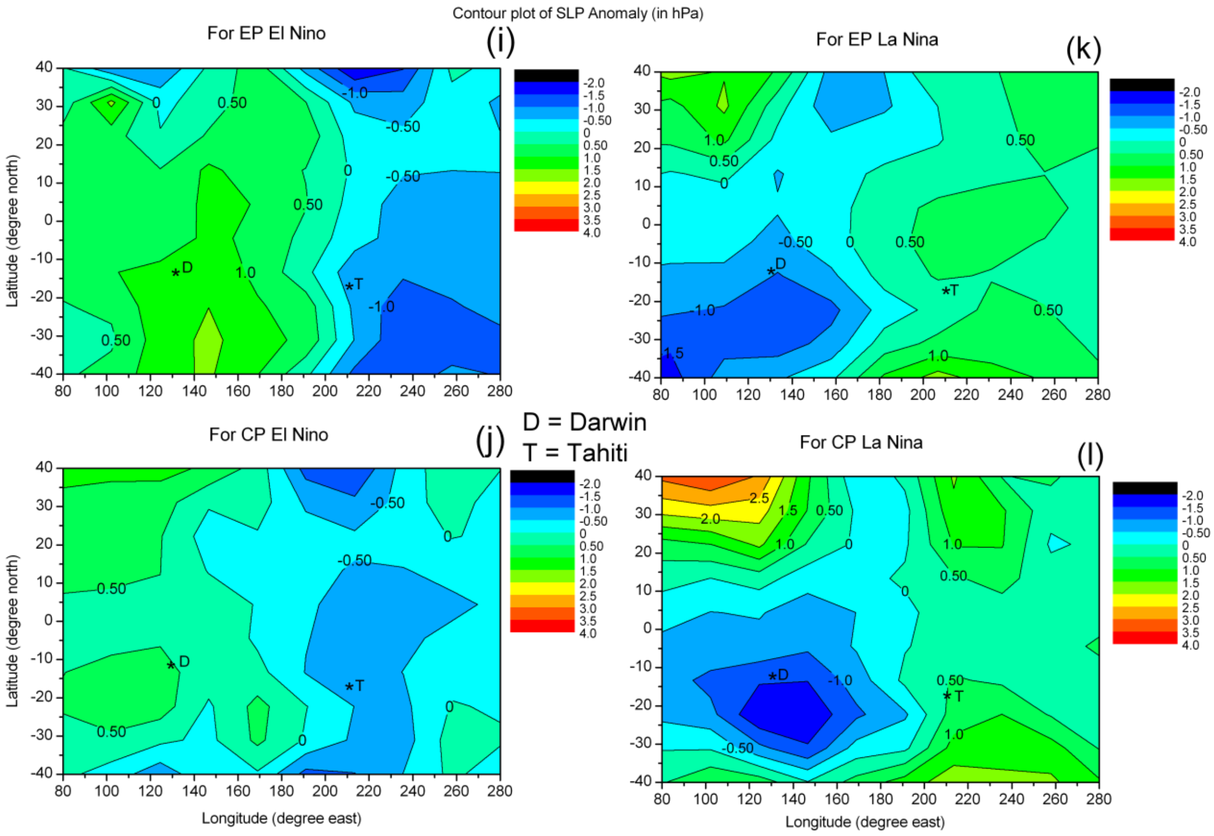

Figure S2 continued………….

Contours of Upward longwave flux anomaly at top of atmosphere (in watts / m^2)

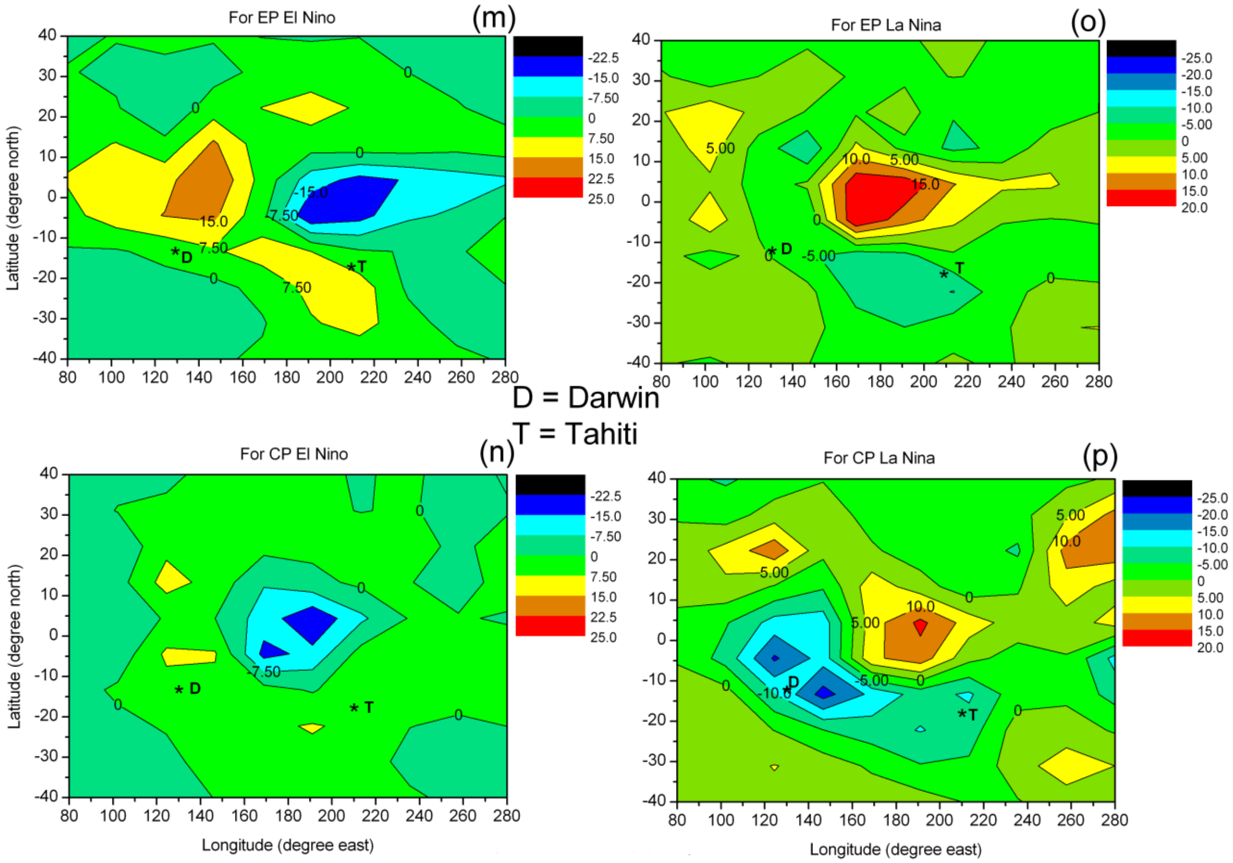

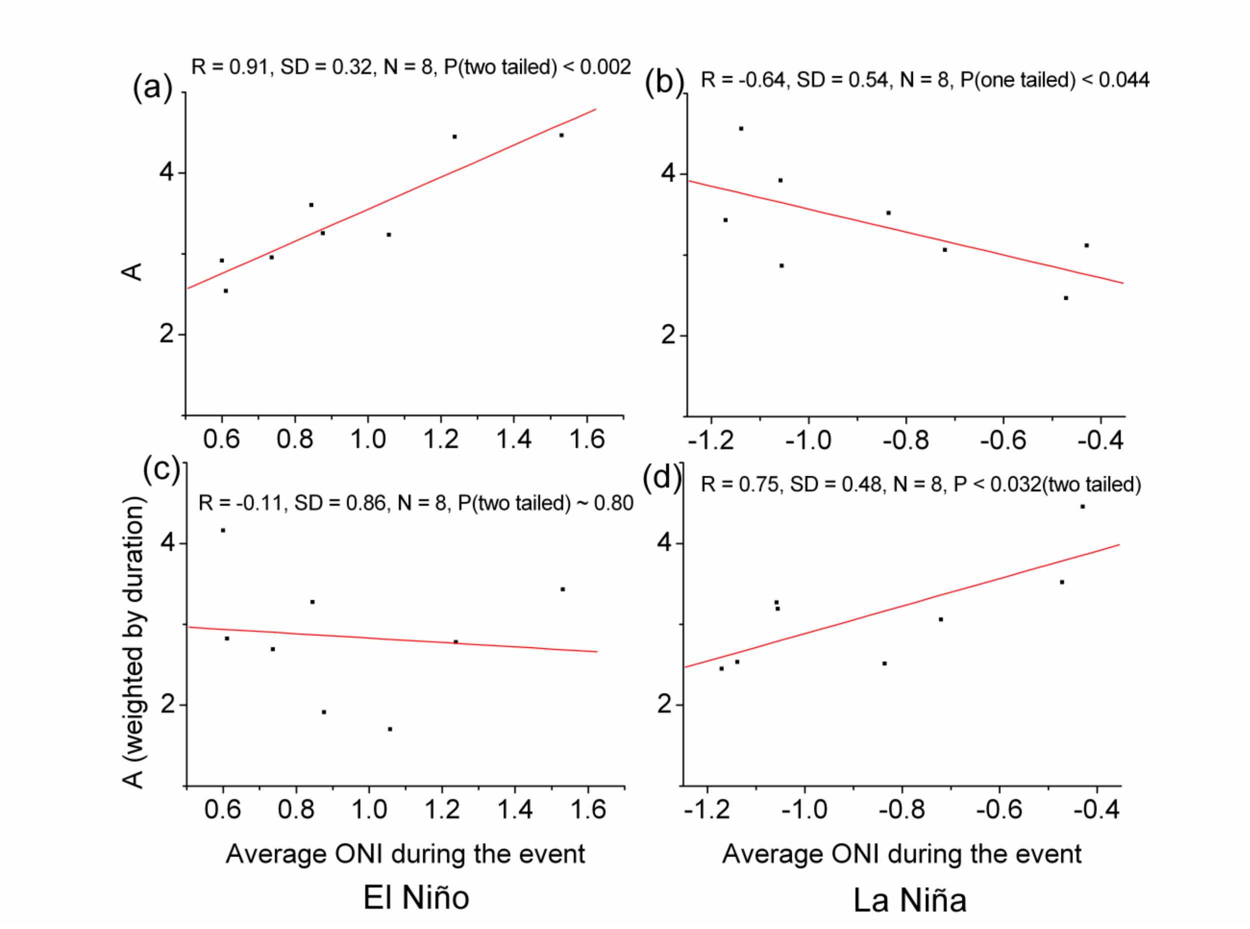

Figure S3

a and b: Scattered plot of the ONI and peak to peak amplitude (A) of the standardized AEI for El Niño and La Niña respectively

c and d: Scattered plot of the peak to peak amplitude (A) of the standardized AEI / Duration (D) of the event for El Niño and La Niña respectively. Origin 8.5 software has been used for making these plots.

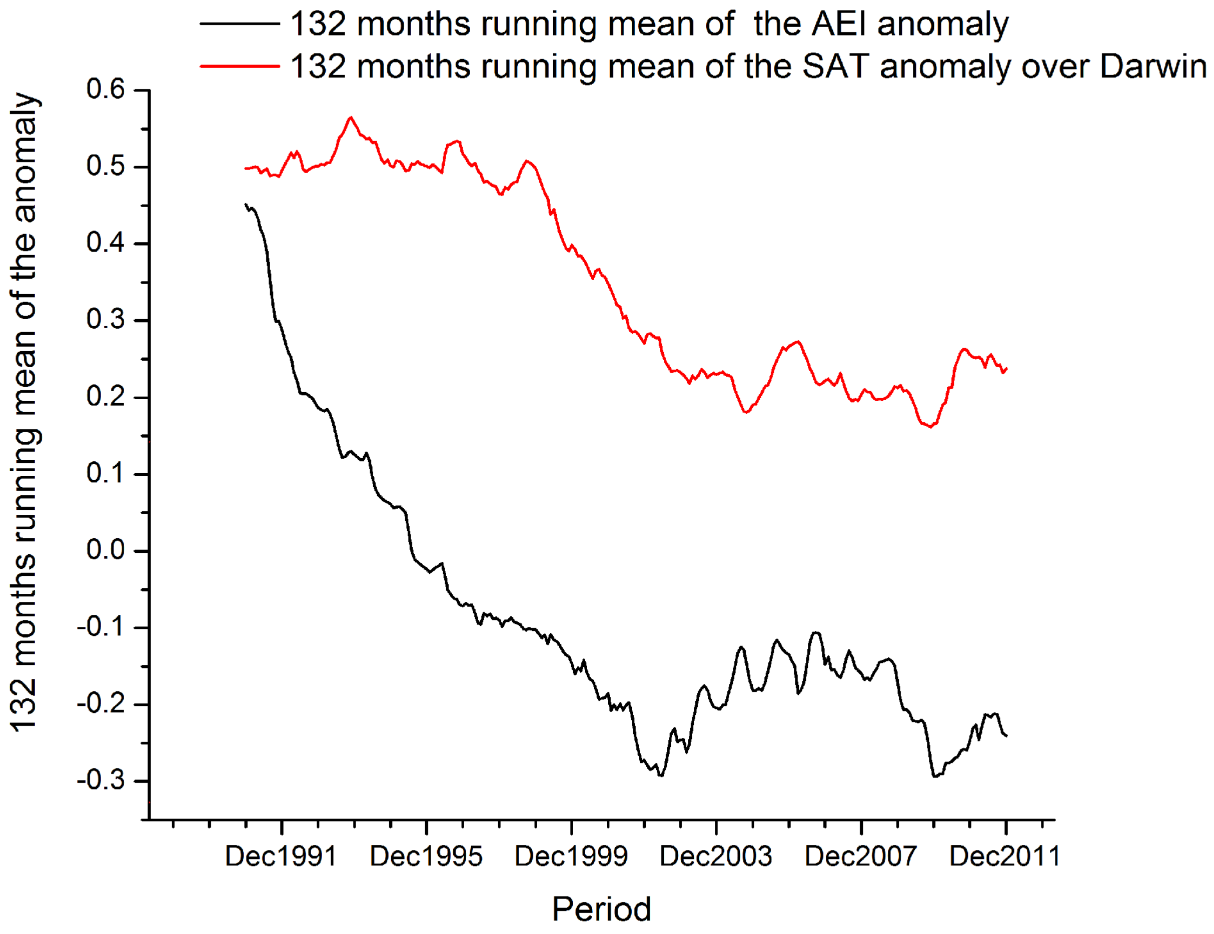

Figure S4: Variation of the adjusted SAT anomaly over Darwin and the corresponding AEI anomaly. Origin 8.5 software has been used for plotting.

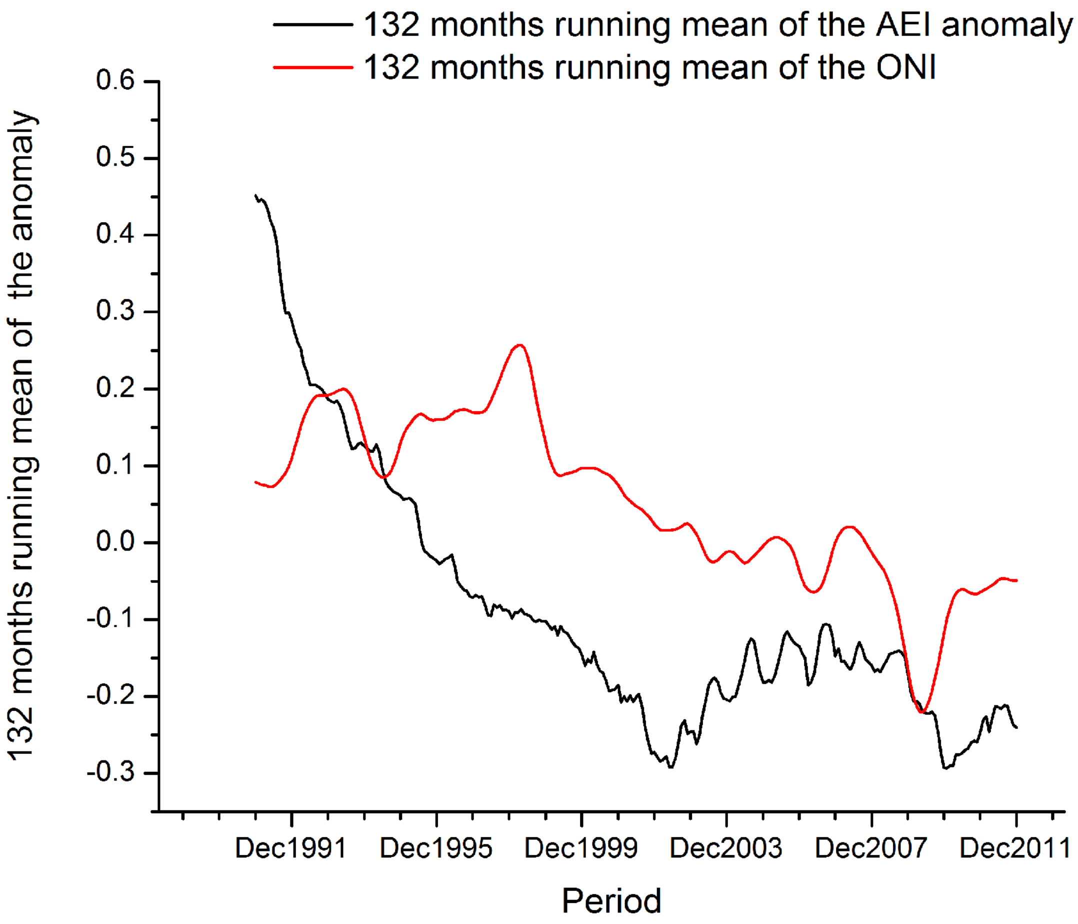

Figure S5: Variation of the adjusted ONI anomaly and the corresponding AEI anomaly. Origin 8.5 software has been used for plotting.

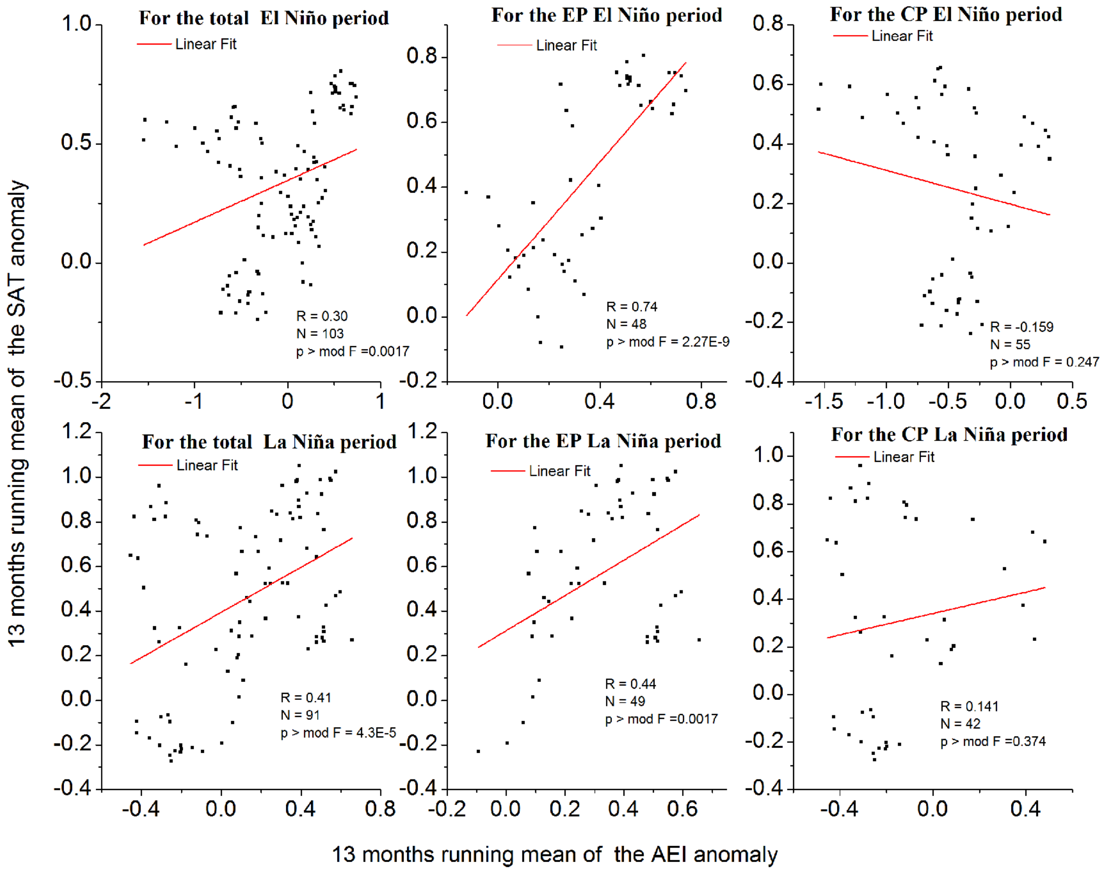

Figure S6: Correlation statistics of 13 months running mean of the AEI anomaly and the corresponding SAT anomaly showing significant correlation for the EP events. 'R' is Pearson's correlation coefficient and N is number of data points and p is the probability showing statistical significance for F distribution. The plots have been made using Origin 8.5 software.

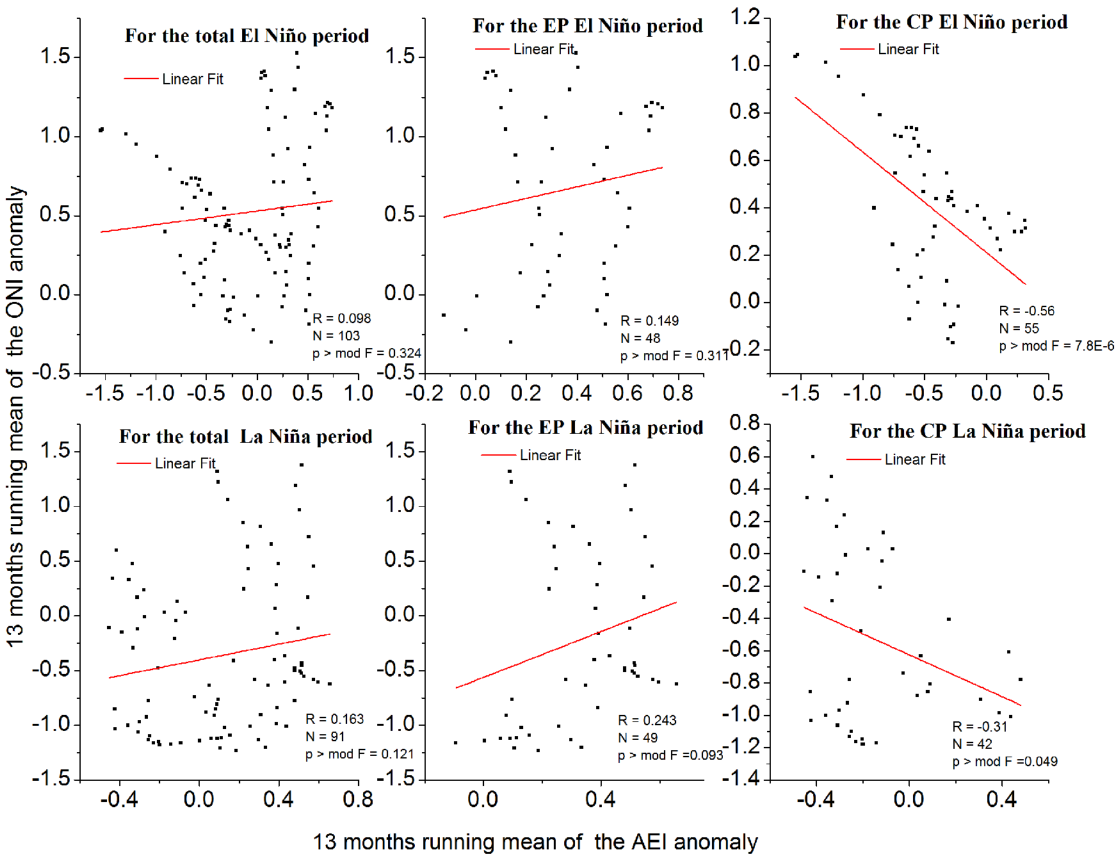

Figure S7: Correlation statistics of 13 months running mean of the AEI anomaly and the corresponding ONI anomaly showing significant correlation for the CP events. 'R' is Pearson's correlation coefficient, 'N' is number of data points and p is the probability showing statistical significance for F distribution. The plots have been made using Origin 8.5 software.

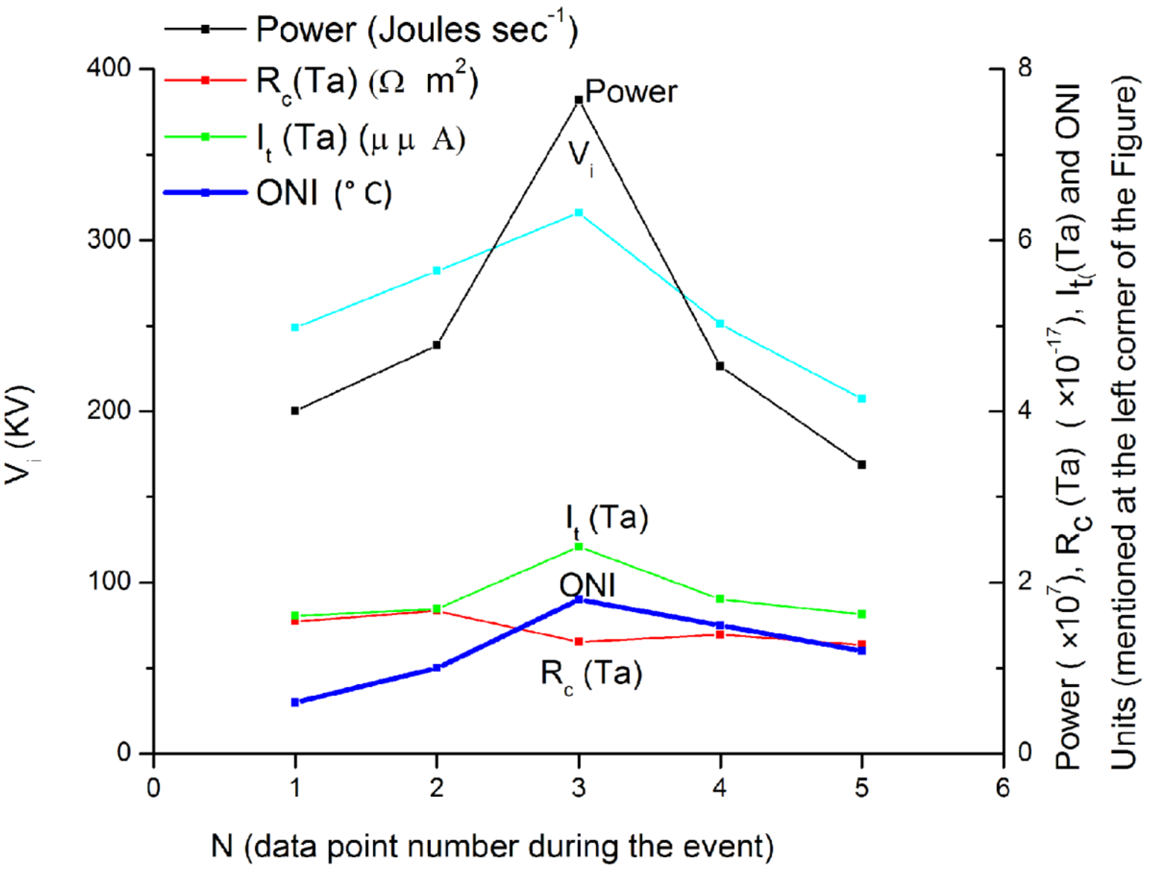

Figure S8: In-phase variation of the measured V_i^1, derived I_t (at Tahiti) and power with the ONI during the El Niño event of April 1982-July 1983. Origin 8.5 software has been used for plotting.

Supplementary Equations

The equations used to calculate atmospheric electrical columnar resistance are as follows:

$R_c = [(H + S) / \lambda(\infty)]$ [2,3] (S1)

where H is the PBLH, S is the scale height of conductivity (= 4340 m) and $\lambda(\infty)$ is the average atmospheric electrical conductivity of the planetary boundary layer.

$\lambda(\infty) = 2en(\infty)k$ (S2)

where e is the electronic charge = 1.6×10^{-19} C, $n(\infty)$ is the calculated steady state concentration of small ions and k is the mobility of small ions, assumed to be equal to 1.2×10^{-4} m^2 V^{-1} s^{-1}.

$n(\infty) = \{-bZ + [(-bZ)^2 - 4\alpha Q(\infty)]^{1/2}\}/2\alpha$ (S3)

where b is a constant involving attachment coefficients between the oppositely charged aerosol and between the charged aerosol and the uncharged particles. $Q(\infty)$ is the ionization at the top of the boundary layer and it is calculated using ionization profile given below (equations S5a-S5d). The values of the recombination coefficient $\alpha = 1.4 \times 10^{-12}$ m^{-3} s^{-1} and that of the constant

$b = 2 \times 10^{-12}$ m^3 s^{-1}.

MODIS AOD values at 550 nm have been converted to those at 500 nm using the following expression[4]:

$AOD_{500nm} = AOD_{550nm} \times (550/500)^{\alpha}$ (S4a)

where α is an Angstrom exponent (440-870 nm)

Total aerosol concentration (Z) is derived from AOD data using the following expression[5]:

$AOD_{500nm} = 0.0027\ (0.36\ Z)^{0.64}$ (S4b)

The average ionization rate ($Q(\infty)$) at the top of the boundary layer is obtained using the following expression[6]:

$$Q(z) = Q_a \, exp\,[(z-z_a)/S_a] \times 10^6 \qquad (z < z_a) \qquad \text{(S5a)}$$

$$Q_a = 15 + 17.778 \, cos^4(\theta) \times 10^6 \qquad \text{(S5b)}$$

$$Q_0 = 1.5 + 0.533 \, cos^4(\theta) \times 10^6 \qquad \text{(S5c)}$$

$$S_a = -z_a / \, ln(Q_0/Q_a) \qquad \text{(S5d)}$$

where $Q(z)$ is the ionization rate at altitude z due to cosmic rays. Q_a and Q_0 are the ionization rates at reference altitude z_a (= 6 km) and at sea level respectively. θ is the co-latitude and S_a is the scale height.

From the calculated $Q(\infty)$ at the top of the planetary boundary layer and derived Z, steady state conductivity $\lambda\,(\infty)$ is calculated. R_c is calculated using (S1).

Supplementary References

1. Markson, R. The global circuit intensity: its measurement and variation over the last 50 Years. *Bull. Am. Meteorol. Soi.*, 223-241, doi:10.1175/BAMS-88-2-223 (2007).
2. Kulkarni, M. N. A new empirical relation to estimate atmospheric electrical columnar resistance, effect of changing aerosol concentration and cosmic ray ionization on it: A quantitative study. *J. Geophys. Res*. **114,** doi:10.1029/2008JD010009,1-13 (2009),
3. Kulkarni, M. N. A new tool for predicting drought: An application over India. *Sci. Rep.* **5**, 7680, DOI: 10.1038/srep07680 (2015).
4. Khan A, Qureshi S., and Blaschke T. Monitoring spatio-temporal aerosol patterns over Pakistan based on MODIS, TOMS and MISR satellite data and a HYSPLIT model. *Atmos Environment.* **45**, doi: 10.1016/j.atmosenv.20011.05.055, 4641-4651 (2011).
5. Andreae, M. O. Correlation between cloud condensation nuclei concentration and aerosol optical thickness in remote and polluted regions. *Atmos. Chem. Phys*. **9**, 543–556 (2009).
6. Makino, M., and Ogawa T. Quantitative estimation of global circuit. *J. Geophys. Res*. **90,** 5961– 5966 (1985).
